# Inaugural Address at the Opening of the Woman’s Medical College of the New York Infirmary

**Published:** 1881-06

**Authors:** M. Putnam Jacobi

**Affiliations:** Professor of Therapeutics


CHICAGO MEDICAL

JOURNAL & EXAMINER.

VOL. XLII.—JUNE, 1881.—NO. 6.

### Original Lectures.

ARTICLE I.

### INAUGURAL ADDRESS AT THE OPENING OF THE WOMAN'S MEDICAL COLLEGE OF THE NEW YORK INFIRMARY, OCTOBER 1, 1880. By M. PUTNAM JACOBI, M.D., Professor of Therapeutics.

LADIES—It is a good plan, on the threshold of any important enterprise, to pause and take a survey of the field we propose to traverse; otherwise we may lose our way, and arrive at the wrong goal at last.

Every enterprise involves difficulties. Difficulties are inseparable from any condition of existence. The question therefore always is, not "Are there any difficulties to encounter?" but "For precisely what difficulties must I prepare?" The difficulties involved in the study and practice of medicine are intrinsic and extrinsic; and we will consider each in its order.

In addition it will be profitable to inquire what especial difficulties attend the study of medicine by women; and, finally, to point out some which we have practically encountered in the working of this school.

The first intrinsic difficulty in medicine consists in the great mass of facts which it is necessary to know, and in the variety of sciences which must be understood in order to interpret these facts. There is a general impression among non-medical people that all medicine can be learned simply by listening to what sick people have to say for themselves; that any one who has listened during a few months or years to such conversations knows all about medicine—is rich in experience; that what such an one does not know is not worth knowing. Now, in reality, such a method would not suffice to teach the pathology of a cold in the head, although a thousand sufferers related the details of their illness with the utmost loquacity. At the very outset of clinical study it is well to be impressed with this fact: namely, that what the patient has to tell you constitutes precisely the least important part of what you must learn about him in order to be able to understand his case, and to do him any good. This is not only true in regard to children, to insane people, to those who are for the time delirious or unconscious, or to those whose willful exaggerations or reticences evidently distort the description of their symptoms. It is true of every one who does not understand the pathological significance of one symptom as compared with that of another: true, therefore, of every one who is not himself a physician. Let us take an individual case—it makes scarcely any difference what. As serving to illustrate many points, I will select a case of fractured skull. The physician is summoned in haste, and learns that an hour previously the patient had fallen from a scaffolding to the street; had been picked up unconscious, and brought home in the same state; that shortly after reaching home he had vomited, but had not, as the saying is, yet come to himself. The physician finds the patient in bed, motionless and insensible. His eyes are closed, but if the lids be raised the pupils will be found to contract, perhaps sluggishly, to the light, and the lids quiver more or less if the conjunctiva is tickled. The breathing is slow and rather labored, and at each respiration the cheeks puff out as if the man were forcibly smoking a pipe. Perhaps from time to time one of the arms is raised and moved convulsively backwards and forwards, then falls again. The face is pale, but when the doctor lays his finger on the pulse he finds no sign of exhaustion; the pulse is full and hard, and rather slow. He will notice that the clothes are wet with urine.

In examining the head he finds on one side, near the vertex, that the hair is matted; and, separating the mass, he comes upon some clotted blood. He presses his finger in the center of the clot, and may find a depression below the level of the cranium. Perhaps when he presses on this depressed portion the convulsive motion of the arm will re-commence. On searching farther, he may notice a clear fluid running from the ear, on the same side with the visible fracture. Here is his case. Now, for the sake of simplicity, I have so stated it that, in regard to the main fact, the doctor is not called upon to make any diagnosis. There is no doubt about it; the man has fallen and fractured his skull. But, before the physician can understand either the extent or the consequence of this injury, he must be extremely familiar with the anatomy of the injured region. He cannot learn this anatomy from looking at the patient, nor at a hundred similar patients. He must have had the opportunity on many dead ones to dissect out all the parts, and study repeatedly their relations to each other. Then only could he know, in the first place, even that there was a brain inside the skull! Further, that the piece of bone which had been driven in by the blow had probably torn the membranes covering the brain, and even the pulpy substance of this vital organ itself. He must remember the sinuses in the membranes, and the effusion of blood that poured out from them was probably now pressing on the surface of the brain. He must be able to tell, in order to furnish the basis for his physiological analysis of the case, just what part of the surface had been injured—the part whose irritation is known to cause convulsive movements of the right arm. He must be able, from his previous knowledge, to trace downwards the direction of an invisible crack, leading from the visible fracture to the base of the skull, and splitting another portion in such a way as to allow of the escape of the clear liquid from the ear. All this knowledge, and that of other details which I omit, must the physician bring to the case from the study of the first science on which medicine reposes—the science of anatomy. He then begins to trace the relation between the symptoms he has observed and the lesion he has discovered, by means of his knowledge of the functions of the parts involved—in other words, by his knowledge of physiology. By a violent shock the functions of important organs have been rudely interrupted. The physician who was not already well acquainted with these functions would be entirely unable to explain why a blow on the head should suspend or alter them. He could not even see any reason for the suspension of consciousness, of feeling, of power of movement, which has been induced by this blow. Still less could he understand the vomiting, the involuntary emission of urine, the convulsive movements of the arm, the puffing of the cheeks, the changes in the respiration and the pulse. In other words, unless he had an intimate acquaintance with the working of the machinery of the body while in order, he would be as little able to understand its disorder as a bricklayer to know why a watch had stopped, or a shoeblack to mend a locomotive. But the analysis of the case is not finished. The fall of a living body from a height is an event not contemplated in the physiological workings of the organism. It is effected according to physical laws, and the fracture of the skull takes place in the same way, and with the same modifications as would a fracture of any inorganic elastic globe. The radiation of the fracture, the effect of the rebound of the head from the pavement, and of the brain within the skull, cannot be studied by the aid of anatomy or of physiology alone; a third science must be invoked—that of physics. Nor is this all. I have spoken of the clear fluid running from the patient's ear. To the uninitiated this would seem to be of much less importance than the blood which matted his hair. But the physician sees in it a symptom of very serious import; he knows that it is a sign of the fracture of a certain portion of the base of the skull, and foretells almost certain death. So much he knows, or should know, as a fact of clinical experience—that is, of the clinical experience of other people; for he ought to be able to interpret this symptom as perfectly in the first case he ever saw as in the fiftieth. To understand exactly what this clear fluid is, he must, however, interrogate something else than clinical experience, for that has interpreted the matter in several different ways. The question has been solved by clinical analysis of the fluid, which has shown that it does not resemble the serum of the blood, which at one time it was supposed to be, but the so-called cerebro-spinal fluid, which bathes the brain and spinal cord, and which cannot be removed, in even small quantities, without the greatest risk to these vital organs. The gravest feature in a case of fracture of the skull is interpreted by means of the science of chemistry.

Here, then, are four separate sciences, with entirely distinct methods, with which the physician must be to a considerable extent acquainted before he can in the least understand the condition of the patient in the case we have imagined: anatomy, or the science describing the form and relative situation of organs; physiology, or the science of the functions of these organs; physics, or the science of the movements of masses; chemistry, or the science of the composition of bodies, including the solids and fluids of the animal organism. When all these have been applied to the problem, the physician is still at the outset of his investigation. It is not enough that he sees, or even correctly understands, the condition in which the patient is; he must be able to foretell the series of changes which this condition is likely to undergo, during its progress towards death or recovery. To do this he must be acquainted with a fifth science—pathology; a science laboriously elaborated from all the experience, the observations, the clinical and post-mortem analyses which have been accumulated during the historical period of the race. Morbid anatomy is properly a branch of pathology, and nothing can be more absurd than the idea that the clinician can busy himself with the sick person during life, and leave to a specialist in "pathological anatomy" the examination of diseased organs after death. You can only properly observe the living sick man when you are able, in imagination, to pierce through the outer coverings of his body, and watch, step by step, the morbid processes which are creeping onward in the recesses of the organism. For this purpose it is essential that a science, really a branch of anatomy, but often regarded as distinct, be assiduously cultivated. I mean the science of histology. It is only when the microscopic structure of the fractured bones and torn membranes is perfectly known that the physician can understand many of the minuter morbid processes whose possibility he foresees—as an osteomyelitis, a meningitis, a capillary apoplexy. Knowing what exists, and also what is likely to occur, the physician is now prepared to intervene to help the patient, and to avert danger as far as this may be possible. In other words, having applied the arts of diagnosis and prognosis, in accordance with the laws of pathology, he is able to apply the art of therapeutics according to the indications furnished, on the one hand by surgery, on the other by the science of the properties of drugs. He will lift up the depressed fragment of bone by means of a trepan; he will apply ice to the head, to keep down hyperæmia of the meninges; he may possibly give bromide of potassium to deaden the activity of the brain when consciousness returns and delirium is imminent.

From this single illustration, you may at once learn several peculiarities of the physician's work. In the first place you have noticed that the knowledge required is not merely considerable in amount but various in kind, and that all these varieties must be co-ordinated into a single conception, which we may entitle knowledge of the condition of the patient. In every step of the physician's career he is obliged to perform this work of co-ordination; obliged not only to know in detail, but to generalize and combine.

Now the capacity for systematic mental combination is essentially a cultured capacity, and a capacity whose effective attainment is a matter of a great deal of difficulty. It is sometimes proposed to evade this difficulty by dividing up medicine into a great number of small sections or specialties, and encouraging every one to devote himself to only one. Even were this done, the difficulty in question would not be removed, but only pushed back a little. Even when a physician professes to attend only to the diseases of a single organ, he still has to do not with one disease but with an entire class of diseases. To decide whether one of *his* diseases exists, he must know enough about a good many others to be able to exclude them from the diagnosis. Or, if he cannot do this, he must get some one else to make the diagnosis for him—that is, to take out of his hands the first large part of his own work. If we suppose these preliminary questions all decided,—and no doubt to remain that disease exists in the organ appropriated by this particular specialist—we still can only understand this by means of a mass of anatomical, physiological and clinical details, out of which he must build up the general conception of the case. Thus the mental operation is the same in kind for the specialist as for the general practitioner.

Specialists are needed for original researches, and to develop the field of medicine in such a way that it may afterward be cultivated by the general practitioner. Auscultation was once a specialty, and only a few physicians even pretended to know how to use the stethoscope. But to day, as you are aware, scarcely any one claiming the name of physician would dare to disclaim his ability to do so.

It will always be desirable moreover, that certain persons endeavor to acquire unusual skill in some particular directions, that they may be called upon occasionally to decide in questions of unusual difficulty. But it must be left to the general practitioner to call in the specialist, as the judge calls an expert into court, to assist in making up the decision. The responsibility of the decision must always rest with the judge, or the physician,—after they have heard all that the experts have to say, and controlled their report by means of their own knowledge of the subject, and general relations of its parts to each other.

There is another way in which a specialist may be called in: namely, like a chiropodist to attend to some entirely subordinate and presumably insignificant detail. Whoever adopts a specialty for the sake of narrowing his knowledge, and not in order to deepen it, is liable to become a specialist of this kind—a mere corn doctor; with no valid claim to membership in a liberal profession.

We return therefore to our assertion that it is impossible for a real physician to escape the necessity of constantly dealing with multiple groups of facts. He cannot therefore be dispensed from the necessity of acquiring the mental culture which alone can enable him to accomplish this task. To further illustrate my meaning, I would point out that there are four successive degrees of generalization that may or must be effected by the physician. The first degree is that which I have already shown to be involved in the very simplest diagnosis of disease in a single organ of the body. In a second degree of complexity the physician is obliged to consider also the co-existence of a morbid condition in some organ, and to ascertain which, if any, are the relations between these two. Thus, if the same patient be suffering from dyspepsia and endometritis, it is very important to know whether the dyspeptic symptoms result from the irritation of the endometritis, or whether the endometritis is the final expression of a state of denutrition originated by the dyspepsia. If, again, a pregnancy complicates the uterine disease, the question of treatment is rendered more difficult by the risks of interfering with the pregnancy.

In a third degree of generalization, the physician must rise to considerations of the pathogeny of disease, and these are inseparable from general philosophic notions to enable him to grasp the theory of the matter. Thus, in investigating a case of phthisis, the physician will go but a little way who rests with the report of subcrepitant râles at the apex of one of the lungs. It is imperative that he understand the theory of phthisis, and the relations between the theory of Bayle and Laennec, which would attribute these râles to ruptured tubercle; the theory of Rindfleisch, which would explain them by the breaking down of masses of tissue chronically inflamed; the theory of Bull, explaining the ulceration process by a dipththeritic-like infiltration. Immediately or remotely, the practical treatment of phthisis is moulded by the theory which may have been adopted.

Similarly, the practical treatment of uterine diseases must vary considerably when the theory of menstruation regards this process as a congestion, or as a plastic process of growth.

The highest degree of generalization is that involved in the pursuit of original researches. Upon this we will not now stop to speak.

Now, as I have already said, the capacity for generalizing is essentially a cultured, an acquired capacity. Whenever it seems to be natural, that is, to come without any special training, it is always wrong. That is to say, untrained persons of active minds, and who are often very ready to generalize, invariably do so from too small a number of facts or data. Hence their conclusions are inadequate or absurd. Homœopathy furnishes an excellent illustration of just this kind of generalization. It has picked up a superficial resemblance between things; has refused to analyze further the real relations of these things, and then insists upon having discovered the true theory of their relations. Thus Hahnemann gives as an illustration of the way in which natural instinct appeals to the law of Similia: the case of a cook who, having burned her finger, plunges it into warm water; or, the boy whose fingers are frost-bitten, yet who takes care to rub them with snow. Now this accidental resemblance between the cause of the injury and the treatment explains nothing. A little deeper examination shows that, in the first case, the warm water is required to relax the distended blood vessels; in the second, the cold is needed to restore the circulation gradually and not with a rush, which might prove fatal to the tissues. In these celebrated examples, an immense fallacy is accepted, by omission of the philosophical distinction between two kinds of causes: the efficient cause, the burn, which has initiated a train of morbid processes; and the proximate cause, that is, the anatomical and physiological conditions upon which the symptoms immediately depend.

To train the mind to handle large masses of facts, it must be gradually accustomed to work with somewhat smaller masses of more accessible facts. This is the reason for that general literary education which, in all European schools, is exacted as an indispensable preliminary to medical study, and which, in this country, is often considered as superfluous. But it can only be so considered by those who have never tried to analyze the mental operations involved in the simplest medical work.

Our illustrative case shows that something else is necessary also. The senses must be trained as well as the mind. I will not now dwell upon the methods for training the senses, but only point out two facts. First, that the facility and accuracy with which the senses work, is largely in proportion to the amount of mental training that guides their operation. You can see, hear and feel a hundred fold more when you know beforehand exactly what is to be felt, or heard, or seen; and when you have an ideal standard with which you can compare the results furnished by your eye, ear, or finger. In the second place, it is logical and much easier to train the senses by means of simple exercises before attempting more complex ones. Thus an excellent preparation for learning how to observe in anatomy, is to pursue observations in botany.

It is now worth while to inquire, since the study of medicine is so vast, what proportion of it can possibly be mastered during a given term of years: in other words, what we may expect a student to know who presents himself for graduation. As the foundation of everything, a really complete knowledge of anatomy is indispensable. It will not do to know that an artery is, as the boy said of Abraham, "there or thereabouts." It will not do to have a general idea that the nerve centers are divisible into a cerebrum or cerebellum, medulla and spinal cord. The anatomical knowledge that is not precise and accurate is as unavailable for the physician as would be a general idea of the county in which a person lived, to the postman charged to deliver a letter to him.

There is another reason for demanding completeness of knowledge in regard to coarse and fine anatomy, and that is, that it is so readily forgotten in after practical life, and requires to be so constantly revived by fresh reference as wanted. Students are apt to think that therefore it never need be fully known at any time. This is a great mistake. What has once been firmly stamped upon the mind, can easily be revived; what has always been vague, will always remain so, unless there take place such a radical change in mental habits and methods as we have no great reason to expect. The science of chemistry, so far as regards its relations to medicine, should also be perfectly known at the outset, and can be known because these medical relations of chemistry are at present comparatively so few. Physiology, on the other hand, embraces a much wider field—more indefinite and more complex details. The knowledge acquired of it during a medical curriculum, must be small as compared with the relative amount attainable in anatomy and medical chemistry. But absolutely, this amount is considerable. It is of the greatest importance that the student learn to distinguish the different degrees of certainty which exist between the various physiological doctrines he hears enunciated. It is in studying them that he is first introduced to the peculiar difficulties of the study of medicine, inherent in its imperfection, in its complexity, and in its progressive character. It is impossible to study physiology by the memory alone. Even to remember its details requires a habit of mental poise—a capacity for criticism and judgment which is only acquired by very careful training. In testing the candidate therefore, we expect to find, not a complete knowledge of physiology, but an *accurate* knowledge of certain fundamental facts, familiarity with accepted methods for both the acquisition and application of physiological knowledge, and some trained judgment in regard to the grouping of facts known; finally, sound and vivid perceptions of the relations of physiology to medicine, and of their constant interdependence upon one another.

Coming now to medicine proper—what may we expect a graduating student to know? It is a mass of knowledge so vast (often *so confused*), so unsystematically grouped together—so largely empirical,—so unequal in its development; its acquisition depends so much upon prolonged clinical experience with personal responsibility, that it is really very difficult to define just how much may be acquired; how much and what must be expected of any one after a given course of study. We can, however, say this: First—That the graduate must be thoroughly acquainted with the rules of diagnosis, and show his ability to apply them in any given case. Second—That he must be acquainted with the typical outline of all classical diseases, and thus know the symptoms upon which the diagnosis is based. Then there will be nothing to prevent him from diagnosticating even the very first case he ever sees of even the rarest disease. Whoever is able to do this; whoever has reached a stand-point from which he can scan the entire horizon of medicine, has reached a beginning whence nothing need prevent indefinite progress. But unless this beginning be reached, the physician is really incapable of making the first decision about any one who comes to his office, or who calls him to their bedside. A doctor who did not know that coryza was one of the symptoms of syphilis, could not safely pronounce with positiveness upon the nature of an apparent cold in the head. Another, who knew of no eruptive fever but measles, would certainly be incompetent to decide that the rash in a given case were not scarlatina or small-pox. No young physician can be expected to know all about all diseases; but he must be acquainted with at least the existence of all that there is to know about. And he must, moreover have attained sufficient mental breadth and grasp to be able to keep the recollection of all firmly and clearly before his mind at the same time. Now this knowledge is really quite attainable by a curriculum of three or four years duration, if the study be sytematically and intelligibly pursued.

The art of therapeutics is much more difficult of acquisition. The treatment of a disease involves many more considerations than even does its diagnosis; and these are susceptible of much greater variety in grouping. Surgical therapeutics, or, as you would perhaps call it, operative surgery, is much the simplest, and, accordingly, is much farther advanced. The logical method would prescribe that before studying the effect of drugs internally administered, the pupil should be carefully trained to watch the effect of the topical applications, the various manœuvers and operations, by which a surgeon deals with cases of external pathology. The question that meets us at the outset is, Is it really possible for us to produce any definite effect upon the processes of a living organism? This question is at present better answered in surgery than in the domain of internal medicine; and we should therefore seek for the answer first there. Yet so easily are we deluded into believing that whatever is familiar is simple, and whatever is unfamiliar is abstruse, that I suppose there is not one of you who would not believe that the action of a dose of castor oil was much easier to understand than the action of a fracture splint; or, again, that any woman physician might be expected, in virtue of her sex, to know something of pessaries, but need not be expected to know anything of orthopœdics, although a pessary for the replacement of a dislocated uterus is strictly a surgical and, by analogy, at least, an orthopœdic apparatus. What we may expect of a student at graduation is, to know the precise physiological action of drugs so far as this is known at present; to know the principal variations in such action occasioned by disease; to know the principal indications for the use of the drugs; and, finally, the principal diseases in the course of which these indications present themselves. It is unnecessary to add that he must know the doses and preparations of these same medicines.

To apply my previous test, I would say that the theoretical possession of this amount of knowledge is quite attainable in three or four years. The practical availability of it, is attainable with such slowness and difficulty, that it would really be desirable to pass a law forbidding any young physician from assuming the full responsibility of prescribing until, for a year, privately or in hospitals, he had practiced under the close supervision of some one else.

The work of co-ordinating multiple facts, which I have said was the characteristic work of the physician, must be begun by the student in his most elementary attempts at mastering knowledge. This is the only way in which he can remember the immense amount of facts he is expected to know. He must bind them firmly into a single bundle, or a definite number of single bundles, or they will all fall apart like scattered sticks.

Every time you learn anything new, you should stop and ask yourselves whether you know everything which is implied in that knowledge. In studying the anatomy of muscles, you have an opportunity of reviving your knowledge of the bones on which they are inserted. In studying the course of arteries and the distribution of nerves you refresh your recollection of the muscles which serve as landmarks to them. In observing any case of disease in the college clinics, it should be your self-imposed duty to ask yourselves if you know all about the anatomy, histology, and physiology of the organs involved in the disease. The constant, faithful, patient repetition of these inquiries would continually render the co-ordination of your various studies more and more easy to you ; would train you in the capacity, invaluable in a physician, of bringing to bear all your knowledge at any given point, and of turning it to account wherever it was wanted. For here is the immense peculiarity of medical knowledge—it must all be turned to account. It is tremendously, often terrifically, responsible. It is this sense of responsibility which should be constantly impelling the medical student to a determination to *grasp* a subject, instead of remaining content to wabble about in it. Medical knowledge is not something which can be purchased and applied to a patient like a plaster or a poultice; it is something to be handled—like a tool, like an ax—and the effectiveness of the handling depends upon the firmness of grip of him who holds the instrument. This fact involves a double responsibility on the part of the teacher. It is not sufficient to expound doctrines and convey information; it is essential to train the minds of the persons who are expected to profit by it. This requires systematic intellectual gymnastics; requires repeated practice in all the mental operations which, in after life, the student will ever be called upon to perform. Thus he must be taught, he must probably be compelled, not only to remember approximately, but accurately; not only to be able to think when at leisure and unencumbered, but under strong pressure, and perhaps in the midst of the most embarrassing circumstances; to express himself, not only in a slovenly, awkward, halting manner, calculated to make nervous people impatient, and timid people alarmed, and arrogant people contemptuous, but in such a clear, concise, forcible way as shall always compel attention and extort respect from the very midst of hostile criticism. The physician, like the soldier, must be trained to act under fire; and a training for mere holiday manœuvers, out of sight of the enemy, is lamentably insufficient for the purpose. Human minds are not pint-pots, into which we may pour water or milk or wine at our option; nor are they often Danaides, which may be quickened simply by immersion in a golden rain from heaven. They are living organisms which can only use what they have assimilated and digested, and wrought into the texture of their inmost fibers.

This vigorous assimilation demands qualities of grit, which are as much moral as intellectual. Many moral qualities are needed in the practice of medicine to meet the difficulties which, though extrinsic to the case considered as an intellectual problem, are very important in its practical discussion. The fundamental difficulty of all lies in the fact that so much depends not only on rigid adherence to rules (and there are many more rules for guidance than you might sometimes suppose), but that nevertheless the final arrangement must be left to the individual tact, discretion, and judgment of the practitioner. The theoretical and practical are inextricably intertwined; and the promptness with which theories will often be found to effect modifications of practice, in itself renders medicine one of the most interesting spheres of human existence. Hence in the most abstract reasoning—if the physician be capable of such—he must always keep his mind intently focussed upon the practical purpose towards which it must converge. He must see all his reasons, not hovering about in the air, like bodiless cherubs, around the bed of his patient; but embodied in tangible facts and and definite actions. He must see that his antiseptic fluids actually reach the infected surfaces; he must see that his hot baths are of a given temperature, and that his cold applications are renewed as often as they grow warm; he must know whether the medicine prescribed has been vomited, whether the food has been given at the stated intervals, whether the pulse has responded to the stimulant. He must know how to enforce his directions, in spite of the reluctance, or indifference, or carelessness, or stupidity, or forgetfulness of his patients; in spite, moreover, of the interference of friends, who invariably try to persuade the sick person to call in another doctor. In many cases the physician must almost, as it were, carry his patient in his arms, encouraging, urging, consoling, inspiring him. To do this he must be capable of sympathy with physical suffering, at once delicate and profound. To be efficacious, this sympathy must be fine, and not blubbering; it must feel for the patient ten times, a hundred times as much as it audibly expresess for him; it must manifest itself in deeds, not in words; in indefatigable efforts to accomplish the essential, not in rambling talk about irrelevant trifles, even when, to the sick person, these seem to be the most important.

And at the same time, while treating his patient as though he were a personal friend—while, if necessary, risking his life for him—the physician must never forget that this same patient is, from the nature of things, a possible enemy. A physician prescribes somewhat as the Spartans under Lycurgus were permitted to propose a new law. If the proposition succeeded, the innovator was honored immensely; but if it failed he was put to death. By the most scrupulous honor and the most conscientious care, the physician is bound to justify a claim to the absolute confidence of his patients; but he must never give them his. He must never be off his guard; never forget that he is the object of incessant criticism, not only for what he does, but also for what he does not do, and for every detail of his way of doing. It is essential that in every detail, in every expression, in the entire mental atmosphere of the physician, the patient should feel himself in the presence of a superior person. He must be conscious that a mind warm, vivid, and penetrating is dealing with his case. He must be conscious, also, that, notwithstanding this personal sympathy, the physician is studying his case as coolly, impartially, abstractly, as if it were a problem in algebra. If he does not do so—if, moreover, he fail to solve the problem—sooner or later the patient will leave him, perhaps with the best good wishes, but still he will leave him, and try his fortune elsewhere.

You see, therefore, that, in order to be a physician, it is not sufficient to have a good memory and be able to pass examinations. This is indispensable, but much more is required. The capacity to examine minutely, yet generalize comprehensively; to take large views, yet not overlook the smallest details; to be quick to notice, yet slow to speak; to reason cautiously, yet decide promptly; to be at once very cool and very warm; to be tenacious of one's reputation, yet indifferent to careless opinions; to be sensitive, yet not touchy; to be patient in temper, yet capable of wrath; to be absolutely honest, yet successfully prudent; to be unworldly, yet capable of managing the forces of the world—all these mental and moral capacities are necessary to enable a physician to study practical medicine, to practice medicine, and to build up a practice out of services rendered to a crowd of sufferers, at once helpless, ignorant, exacting, and capricious. Varied as are the mental and moral capacities required for this enterprise, they may be all traced back to three, namely: Ability to think, character to control, and honor to act from an internal instead of an external standard of obligation. When these qualities are not possessed, or have been insufficiently developed, one of two things happens. Either, in the competitive struggle, the ill-prepared physician gets crowded out by more capable rivals; or else, he manages to hold his place, but at the expense of patients, ill-treated by him, and who might have been better treated by some one else.

These patients are the persons who must be kept in view by the examining boards, who are licensed with the power to grant medical diplomas. This power constitues a tremendous social responsibility. It is quite possible for a medical college to have no other function than that of testing candidates. This is the case with the University of London. It gives no instruction at all, but it grants degrees to all persons, who, having been educated elsewhere, are able to pass the scrutiny of its examiners. It must always be the principal function of a medical college to fix the standard of attainment;—and to point out what must be learned and what must be done to reach this: and thus, finally, ascertain as far as possible whether candidates have fulfilled these conditions. The college is then able to turn to society and say to people entirely helpless to judge for themselves: "Here is a person to whom, in perfect confidence, you may entrust your most important interests. Upon his knowledge, his skill and judgment you may rely as completely as upon that of any one of equal professional age in the profession; and upon his honor, you may at once rely absolutely." The responsibility attaching to this assertion is so tremendous: the consequences of a false assurance of confidence may be so various and so disastrous, that in comparison with it, sympathy for the disappointment of an unprepared candidate ought to be left entirely out of sight. The examining board betrays its social trust the first moment that it consents to confer a certificate of capacity upon an incapable person. In such a case, it becomes culpable of the same crime, for which, after the recent Seewanhaka disaster, the grand jury indicted the inspectors to whose false assurances of security that terrible disaster was traced.

This consideration comes up with especial force in regard to women medical students. These are still, by the majority of the public, regarded as disqualified from the practice of medicine merely by reason of their sex. The same reason is not always given. It is sometimes alleged that they have too little mental capacity; sometimes too little general education; sometimes too little physical health; sometimes that their judgment is too flighty; sometimes that their temperament is too excitable; sometimes that they have too little self-reliance; sometimes that they have too much self-assurance. But that, whatever be the reason, they are intrinsically unable to make, or to be made into, safe practitioners.

When you have assembled together in an institution legally chartered and recognized by the State for instruction in medicine; when you find yourselves going through the same exercises as those which are being carried on in every other college in the city: ultimately brought to a commencement hall, where a band of music and a valedictory address seem to imitate to perfection those of the best equipped universities, it is not unnatural for you to feel as if all this vexed question about women's capacity for the profession of medicine had been entirely settled. In reality, however, it is not so. It has almost reached the point where it can be decided on its real merits, and on the actual results of the work done by women as physicians. But it has not quite reached even this point, since the preparation afforded to the mass of women students is still inferior to that which is attainable, if not attained by men. In the meantime, although skepticism has become more polite, or veiled, it is still much more wide spread than you would probably imagine. Only a few months ago a prominent physician of this city expressed the doubt,—in private conversation it is true,—whether, in twenty-five years from now, any women would be found practicing medicine. A professor of Ann Arbor has recently written two letters to a Michigan paper to express himself as "decidedly adverse" to the attempt of women to practice medicine. A few years ago, one of the lady trustees of this college told me that a friend of hers asked her why she had anything to do with women doctors, when it was notorious that they were all Free Lovers. Last year another lady trustee explained the indifference of so many influential people to the success of this school, as compared with their interest in the Training School for Nurses, on the ground that the latter were felt to be a necessity, while a medical school for women could only add a poorer class of doctors to an already over-crowded profession. There were more doctors turned out now every year than could find work to do in the community; there was not really any reason for helping to manufacture more. When I suggested that some of the women doctors were expected to displace a certain number of men, she was perfectly astonished.

She tacitly took for granted that all the men must first find something to do: what was left over only, could be taken up by the women.

But now this is the very point at issue. Since society is, numerically speaking, already supplied with quite enough doctors, the only way in which women physicians can possibly gain any footing is by displacing a certain number of men. In order to do so, they must evidently show qualifications superior to those of the physicians whom they displace, and sensibly equal to those of the physicians with whom they are to be ranked on an equality.

Now, it is well to at once recognize the fact that a good many difficulties stand in the way of both achievements, and these can only be surmounted when they have been distinctly recognized and systematically provided for.

It is very difficult for women to make headway against the settled opinion of society that they are unfit for final responsibilities. This opinion not only often hinders their education to responsibilities, by preventing people from entrusting them into their hands; but it reacts upon their own minds, is liable to make them hesitating, undecided, timid, and thus still more to justify the social prejudice. It is a common remark, "Women do not feel any confidence in women; in an emergency, they must always appeal to men." This, because it is the habit of centuries so to appeal; because the mass of knowledge, power, and force is still overwhelmingly on the masculine side; because, perhaps, the mass of such force always will be so distributed, and the women in positions of first-class responsibility will always be sufficiently in the minority to be deprived of the benefit of traditional influence and prestige. The claim to equal confidence as made by a woman must be a peculiarly intellectual one, because it must be sustained in spite of a conspicuous inferiority of physical strength. To produce upon the mind of the average public the same impression as may be made by a masculine physician, the woman must exhibit comparatively more force of mind and character, because the force of body is so much less, and in a question of forces the impression unconsciously received from physical size must be taken largely into account. It is like a watch as compared with a locomotive; if there be not greater precision of action in the one, to balance the imposing massiveness of the other, the more delicate instrument must be crushed with contempt. Many mental habits of women stand in the way of their acquiring this superior precision and surety. These can only be acquired by means of repeated tests, and by the prompt rejection of all work which does not come up to a given standard. But women, as a class, are never habituated to test their work ; and have an almost irresistible tendency to appeal to some personal influence to avert the consequences of its failure. I do not wish to make any protest against the habit of appeal to personal influence ; it is ingrained in the nature of things and of women, and when restrained within its proper sphere does a great deal of good. But it certainly has a tendency to deteriorate the character of women's work, unless they strenuously resist it.

In the general theory of society, women are not expected to achieve anything. This theory is sometimes the reason that they are not trained to achieve anything—that their education is so flimsy and scrappy ; sometimes, again, on account of this theory, so much surprise is elicited when they do achieve ever so little, that they are flattered into a very dangerous over-estimate of their own powers. In this flattery there is often concealed the feeling expressed by Dr. Johnson in his celebrated remark about a woman preaching : "It is," he said, "like a dog standing on its hind legs —it is not well done ; but then the wonder is that it is done at all." The tendency of women to nestle within a little circle of personal friends, and to accept their dictum as the ultimate law of things, renders them as liable to be spoiled by this sort of admiration, as they are liable to be discouraged when they do not get any admiration at all.

The remedy for all this, however, is not hard to find. A woman must accustom herself to dispense with the personal approbation of the people she knows, as a stimulus for exertion. She must learn to work for the sake of the work ; she must be ready to put into it an amount of labor as would not "pay" if estimated merely by what can be seen on the surface ; she must know how to hold her own standard a good deal higher that of partial friends ; she must learn, not only to keep calm under blame, but, what is much more difficult for a woman, to bear praise unmoved, otherwise she will soon cheapen with the praise. The careful self-education of women in all these matters is so much the more important, because it is only by means of it that they can hope to overcome the more external difficulties by which they are weighted. It will not do to forget that their health is often fragile; that they often begin to study somewhat late in life, and when much needful vitality has been exhausted; that they are more frequently involved in family responsibilities and complications. At any rate there is always one two-fold dilemma. They are either pecuniarily well off, and then the force of tradition tends to keep them from working, because, as it is said, there is no occasion for it; or else they work—they study medicine, for instance—under such pressure of pecuniary necessity as leaves them barely the time or the means for adequate preparation. It is comparatively rare that the happy mean exists, where the student possesses just enough money to secure her from want, yet not enough to take away the stimulus for exertion. This is exactly the amount required.

The question of marriage again, which complicates everything else in the life of women, cannot fail to complicate their professional life. It does so, whether the marriage exist or does not exist, that is, as much for unmarried as for married women. In my opinion the increased vigor and vitality accruing to healthy women from the bearing and possession of children, a good deal more than compensates for the difficulties involved in caring for them, when professional duties replace the more usual ones, of sewing, cooking, etc. But in this delicate and important matter the facility of adjustment will vary in every individual case. Many married women will lose all interest in medicine as soon as they have children, as many now fail to develop the full needed interest precisely because they have no other, and are dispirited by isolation from family ties. Many will interrupt their practice during the first few years after marriage to resume it later. Whatever is done, either with or without marriage, can evidently be well done only in proportion as more complete intellectual development and more perfect training enables the woman to cope with the peculiar difficulties inherent in her destiny.

Women may be said to have obtained a foothold in medicine in modern times on account of the sudden development of gynæcology. It cannot be said that women have contributed much towards this development; but in the treatment of uterine diseases the desirability of women physicians from motives of delicacy, becomes so evident, that a powerful impulse has been created in favor of allowing them to practice at least this branch of medicine. From what I can learn, the majority of women who study medicine do so with the expectation of at once becoming specialists: and certainly, the majority of persons who think of consulting them, think of them first and foremost, if not exclusively, in this connection.

Now, nothing can be more certain than, if women are enabled to practice medicine only in this specialty and for this reason of delicacy, they must, sooner or later, be again excluded from medicine altogether. I say again, because as you know or should know, women have at many different times been admitted to the privileges of medical studies and practice, but have never gained so firm a footing that they were not liable to be displaced. The motive of delicacy; the motive of self-support; the motive of desire for wider spheres of action, are all perfectly legitimate motives, but they are extrinsic to the real reason for the existence of any class of practitioners. This reason is, that such a class is in possession of knowledge which enables it to understand disease, and to cure the sick, and which justifies its members in assuming full responsibility. This full responsibility cannot be assumed, except after liberal study of the whole field of medicine. If, at present, here and there a specialist may arrive at distinction who really only knows one thing: he can only do so because the mass of the profession know a great deal more. If an entire natural class of people devoted themselves exclusively to one thing, they would soon not know even that. Instead of obtaining a position superior to that of the rest of the profession, they must sooner or later sink to an inferior one. In the case of gynæcology and women, the practical experiment has been made: the services of women have been sought on a large scale exclusively from motives of delicacy, and you know in what way. The women were merely assistants—employed to make uterine examinations and report to physicians who were strictly forbidden to make such examinations themselves. The women experts learned as little of the subject as Milton's daughters did of the Latin they read to him without understanding it. The progress of science was retarded, and their intervention was finally discarded as cumbersome. If women will use this specialty, now often thrust upon them, as a stepping-stone to general medicine; if they will look upon it as the small end of a wedge, and persist in driving it forward to a larger end; then they may assure their position, and that of their successors, by means of this temporary opportunity. But if they do not obtain a foothold on the broad, intellectual basis of general medicine; if they content themselves with claiming this little corner, they will never really gain a high place even there: they will be driven out, little by little, until at last the gynæcological wave may pass by, and leave them stranded. There may be less liability to uterine diseases; or these may be so much more easily foreseen and prevented that much less "local treatment" remains to be instituted; or the sentiments of delicacy may change. Just imagine what would become of a class of physicians now-a-days who had devoted themselves exclusively to the treatment of scurvy or of leprosy! Their occupation would be gone with the disappearance of the disease; and the boon to humanity would result in ruin to their class.

I wish now, in concluding, to call your attention to a last class of difficulties, especially connected with medical schools for women. These difficulties all arise out of one fact, namely, that there are not as yet a large enough number of women studying medicine to support medical schools on a large scale; and schools on a small scale are inadequate, because there is is no such thing as large or small in medicine.

During the thirty years which have elapsed since women first began to study medicine in America, there have always come forward a much larger number to claim the right to practice than to crave the privilege of being thoroughly well educated. This unfortunate majority has been the cause of immense injustice to the higher toned minority, because they have constantly tended to drag the conditions of medical education down to the level of their capacity, or intention to fulfill them. The competent have often been sacrificed, in order that the incompetent might be satisfied. A Nemesis never fails to wait upon inefficient intellectual work. It invariably grows lifeless, dull, uninteresting; it finally ceases from sheer inanition. On the contrary, nothing more is required to quicken any subject or any occupation into the most vigorous life and fertile interest, than that every one engaged in it should be inspired with an ardent desire for knowledge and for high attainment. Whenever people are content to do a thing in a slovenly and wanton manner, they very soon get to the end of it. But whenever they try to do it as well as it is possible to be done, or try to learn everything about it that any one else knows, they find themselves at the beginning of a task to which there is no end. They find more to do every day; every day, also, they find more power to do it.

If all the students of this, or any other school, were thoroughly imbued with the determination to accomplish the work before them in the best possible manner, many of the difficulties inherent in the comparative smallness of the school would vanish. You should learn to look at yourselves as a colony just landed in a new country; compelled to found a state in spite of hardship, and peril, and danger, and isolation, by means of the vigorous and intelligent co-operation of each of its members. I do not know that any more instructive reading can be found than the history of colonies, a theme with which every American certainly should be thoroughly familiar. In studying the various destinies of the early settlements of this country, you may gather many hints of importance applicable to our present situation. For us, also, the sea has been traversed, the landing effected, the howling savages, represented by the medical students, temporarily repelled. But that is about all which has as yet been done. It remains to be seen whether our colony contains in itself the stuff out of which the Bay State was built up; or rather those vicious and corrupting elements which corroded to destruction so many settlements south of the Potomac. And do you know what was the one predominating influence that led to such destruction? It was that the mass of gentlemanly emigrants, who had not learned how to dig, and who were by no means ashamed to beg; who had left the mother country, not to seek an opportunity to work more, but to work less; to shirk all the work they possibly could; to profit by the industry and courageous patience of their companions, in order to share, without due share of labor, the revenue accruing from their tobacco and their corn. These are not the characters which could have founded Massachusetts and laid the corner-stone of that State, where, a century later, "embattled farmers could fire the shot that echoed round the world."

Their's is the stuff, these are the characters, this is the austere, self-denying, intelligent heroism, which is needed for our enterprise —for this also still deserves to be called heroic.

---

